# Auditory Evoked P300 Potential in Patients With Parkinson’s Disease

**DOI:** 10.7759/cureus.45933

**Published:** 2023-09-25

**Authors:** Dhivya Rajendran, Rajiv Bandhu, Sujata Gautam, Rajinder K. Dhamija, Sunita Mondal

**Affiliations:** 1 Department of Physiology, Lady Hardinge Medical College and Associated Hospitals, New Delhi, IND; 2 Department of Neurology, Institute of Human Behaviour and Allied Sciences, New Delhi, IND

**Keywords:** parkinson's disease, idiopathic parkinson's disease, idiopathic parkinson's disease (ipd), moca score, cognition in parkinson's disease, p300 potential

## Abstract

Background: Parkinson’s disease (PD) is the second most common neurodegenerative disorder. Though the cardinal features of PD are motor symptoms, it is also associated with many non-motor symptoms, such as cognitive impairment, autonomic dysfunction, sleep disorders, and depression, which could affect the quality of life. Early identification of PD's non-motor signs can aid in the diagnosis of PD. The current research aimed to assess the neurophysiological changes in PD patients using auditory evoked P300 potential and to determine the possible correlation between P300 wave components and cognitive impairment.

Materials and methods: This cross-sectional research involved 32 idiopathic PD patients. The neurophysiological changes in PD patients were studied using auditory evoked P300 potential and the obtained data were compared with normative data. The patient’s cognitive status was scored using the Montreal Cognitive Assessment (MoCA) questionnaire and they were divided into two groups: the patients with normal cognition and the patients with impaired cognition.

Results: The participants showed a significant decrease in P300 amplitude (p = 0.000) but no change in P300 latency when compared to normative data using the Wilcoxon rank sum test. Also, there was a positive correlation between the MoCA score and P300 amplitude (p < 0.05), indicating that if cognition is impaired, P300 amplitude would also be reduced. There was a significant difference between PD patients with impaired cognition and patients with normal cognition in the P300 amplitude at Cz (p = 0.001) and Fz (p = 0.003) when the Mann-Whitney U test was used. These findings indicate that it is possible to notice changes in the P300 wave components among PD patients when their cognition is impaired.

Conclusion: Auditory evoked P300 potentials can be used to objectively evaluate cognition in PD patients and by starting supportive therapy, the quality of life for PD patients can be improved.

## Introduction

Parkinson’s disease (PD) is the second most common neurodegenerative disorder, with a global incidence range from five to more than 35 cases per 100,000 individuals yearly, with a prevalence of 0.3% [1]. The pathologic hallmark of PD is characterized by depigmentation of the substantia nigra and locus coeruleus with neuronal loss in the pars compacta of the substantia nigra. Due to this, the basal ganglia experience a dopamine shortage, which causes a movement disorder, characterized by classic Parkinsonian motor symptoms that include bradykinesia, rigidity, resting tremor, and gait with postural instability [2,3]. Not only dopaminergic neurons but also serotonergic neurons in the raphe nuclei, cholinergic neurons of the nucleus basalis of Meynert, norepinephrine neurons in the locus coeruleus, as well as neurons of the olfactory system and autonomic nervous system are all influenced by neuronal degeneration with inclusion body formation. This nondopaminergic pathology most likely led to the development of the non-motor signs of PD [4].

According to a number of studies, non-motor symptoms such as cognitive decline, autonomic dysfunction, sleep issues, depression, and hyposmia can develop years before clinical motor symptoms [4]. By identifying PD's non-motor symptoms earlier, PD can be diagnosed. Cognitive impairment is increasingly recognized as the most common non-motor sign of PD. It often manifests in the early stages of the disease and may be related to the subcortical lesion of the brain [5]. Hence, we focused more on the assessment of these cognitive functions in PD patients using the Montreal Cognitive Assessment (MoCA) questionnaire and event-related potentials (ERP).

ERP is an objectively non-invasive approach for studying information processing and cognitive brain functions, such as attention, learning, memory, and decision-making, characterized by their positive or negative polarity, latency, and high temporal resolution [6-8]. It is commonly obtained by using an oddball paradigm when a subject perceives an infrequent target stimulus in a stream of often occurring standard stimuli. The P300 wave occurs when the subject is actively involved in the task of detecting the targets. The P300 latency alters with the difficulty of discriminating the target stimulus from the frequent stimulus and the amplitude varies with the improbability of the targets [9].

The latency of the P300 wave was significantly delayed in studies by Sarikaya et al., Lopes et al., and Tokic et al. among PD patients, respectively [2,10,11], while it was not prolonged in studies by Raudino et al., Toda et al., and Prabhakar et al. [12-14]. Also, it was noted by Tokic et al., Tang et al., and Raudino et al. that PD patients had a considerable drop in the amplitude of the P300 wave [2,12,15]. Numerous studies have been conducted using P300 components to evaluate cognitive impairment in epileptic patients, PD patients, and patients with psychiatric disorders [16]; however, consistent conclusions have not been reached, and there have been few studies, particularly in the Indian context, regarding neurophysiological assessment in PD patients using P300. Thus, the current study aimed to assess the neurophysiological changes in PD patients using auditory evoked P300 potential to determine the possible correlation between P300 components and cognitive impairment, which was assessed by using the MoCA questionnaire.

## Materials and methods

Subjects

The present study was a descriptive cross-sectional study conducted in the Department of Physiology in association with the Department of Neurology, Lady Hardinge Medical College and Smt. SK Hospital, New Delhi, after obtaining Institutional Ethical Committee approval (LHMC/IEC/2020/PGThesis/132).

After receiving informed written consent, 32 patients with diagnosed idiopathic PD using the clinical diagnostic criteria of the UK Parkinson's Disease Society Brain Bank [17] were enrolled in the study between January 2021 and June 2022 from the movement disorder clinic of the Department of Neurology.

Exclusion criteria include parkinsonism plus syndrome patients, secondary parkinsonism patients, psychiatric illness patients (anxiety, depression, schizophrenia), patients who are taking medications affecting cognition, patients with a history of neurosurgery or having cognitive impairment by other causes (seizures, stroke, head trauma), heavy smokers, alcohol/ drug abuse patients, presence of any significant hearing impairment.

Procedures

The participants underwent a hearing assessment using tuning fork tests on the day of OPD, and a proforma with all the pertinent information like disease onset, disease duration, treatment details, investigation reports, personal habits, family history, and disease severity score was filled out. The patients were instructed to report to the physiology lab on the specified date and time (around 10.00 AM), to record P300, with proper pre-recording instructions.

On the day of the P300 potential recording, a MoCA questionnaire was also completed to evaluate the patient's level of cognition. The MoCA questionnaire is a quick screening tool for mild cognitive dysfunction. It evaluates various cognitive abilities, including executive functions, memory, language, visual-spatial skills, conceptual thinking, arithmetic, and direction. It also measures attention and concentration. The administration of the MoCA takes about 10 minutes. There are a total of 30 possible points and a score of 26 or more is regarded as normal [18].

P300 recordings were done per the International Federation of Clinical Neurophysiology (IFCN) guidelines using the Schwarzer Topas electromyography (EMG) neurophysiological measuring system (Natus Europe GmbH, München, Germany), a four-channel EMG/nerve conduction study/evoked potential system. Recordings were taken in a sitting posture after explaining the procedure to the patients in a noise-free environment using a standard auditory oddball paradigm stimulus. Surface electrodes were placed according to the 10-20 international system at the points Fz, Cz, Pz, and mastoids with a forehead ground. The subjects were instructed to concentrate on the target stimulus in a stream of standard stimuli and count them. They were asked to report the total target stimuli at the end of the session. The signal is picked up by the electrodes, filtered, amplified, averaged, and displayed on the screen. The peak latency and base-to-peak amplitude of the P300 wave were measured from the screen [19].

Statistical analysis

The data obtained were tested for normal Gaussian distribution by using the Kolmogorov-Smirnov test and Shapiro-Wilk tests for normality and revealed to have a significantly skewed distribution (p = <0.05). For comparing medians between obtained data and normative data [10], the Wilcoxon rank sum test was used. To correlate the MoCA scores with the latency and amplitude of the P300 wave, the Spearman correlation test was used. Mann-Whitney test was used for intergroup comparison (PD patients with cognitive impairment vs. patients with normal cognition). A P-value of <0.05 was considered significant.

## Results

Table 1 lists the demographic information and clinical traits of PD patients. Participants in the study had a median age of 60 years, an IQR of 13 years, and a male-to-female ratio of 1.5:1. According to the MoCA score, 25 out of 32 PD patients had cognitive impairment, and nine patients also had comorbid conditions.

**Table 1:** Demographic and clinical characteristics of Parkinson’s disease patients MoCA: Montreal Cognitive Assessment.

Variables	No. of participants, n (%)
Gender	
Males	20 (62%)
Females	12 (38%)
Age in completed years	
40-49	5 (16%)
50-59	10 (31%)
60-69	12 (37%)
70-79	5 (16%)
MoCA score	
Cognitive impairment	24 (75%)
Comorbidity	
Hypertension	4 (13%)
Diabetes	3 (9%)
Hypothyroid	2 (6%)

The P3 wave's median latency at Pz, Cz, and Fz was 354 ms, with IQRs of 65 ms, 70 ms, and 63 ms, respectively. The latency of the waves was within the normal limit when compared to normative data using the Wilcoxon rank sum test. At Pz, Cz, and Fz, the median amplitude of the P3 wave was 3.5 mV with an IQR of 3 mV, 3 mV with an IQR of 3 mV, and 2 mV with an IQR of 3 mV, respectively. The amplitude of P300 waves was significantly reduced (p = 0.000) at all locations when compared to normative data using the Wilcoxon rank sum test (Tables 2, 3).

**Table 2:** Comparison of P300 latency with normative data

	Median P300 latency (IQR) of study participants	Median P300 latency of normative data	p-value
Pz	354 (65)	346.2	0.079
Cz	355 (70)	346.9	0.661
Fz	356 (63)	346.5	0.601

**Table 3:** Comparison of P300 amplitude with normative data ** denotes highly significant.

	Median P300 amplitude of study participants (IQR)	Median P300 amplitude of normative data	p-value
Pz	3.5 (3)	9.6	0.000^**^
Cz	3 (3)	9.2	0.000^**^
Fz	2 (3)	6.6	0.000^**^

The relationship between the MoCA score and the P300 wave components is depicted in Table 4. MoCA score and P300 amplitude were found to be significantly positively correlated at Pz (p = 0.000), Cz (p = 0.001), and Fz (p = 0.049); however, P300 latency and MoCA score were not significantly correlated.

**Table 4:** Correlation between MoCA score and P300 wave components MoCA: Montreal Cognitive Assessment. * represents significant; ** denotes highly significant.

	MoCA with P3 latency			MoCA with P3 amplitude		
	Pz	Cz	Fz	Pz	Cz	Fz
Correlation coefficient	0.110	0.191	-0.099	0.772	0.550	0.351
p-value	0.549	0.295	0.590	0.000**	0.001**	0.049*

**Figure 1:**
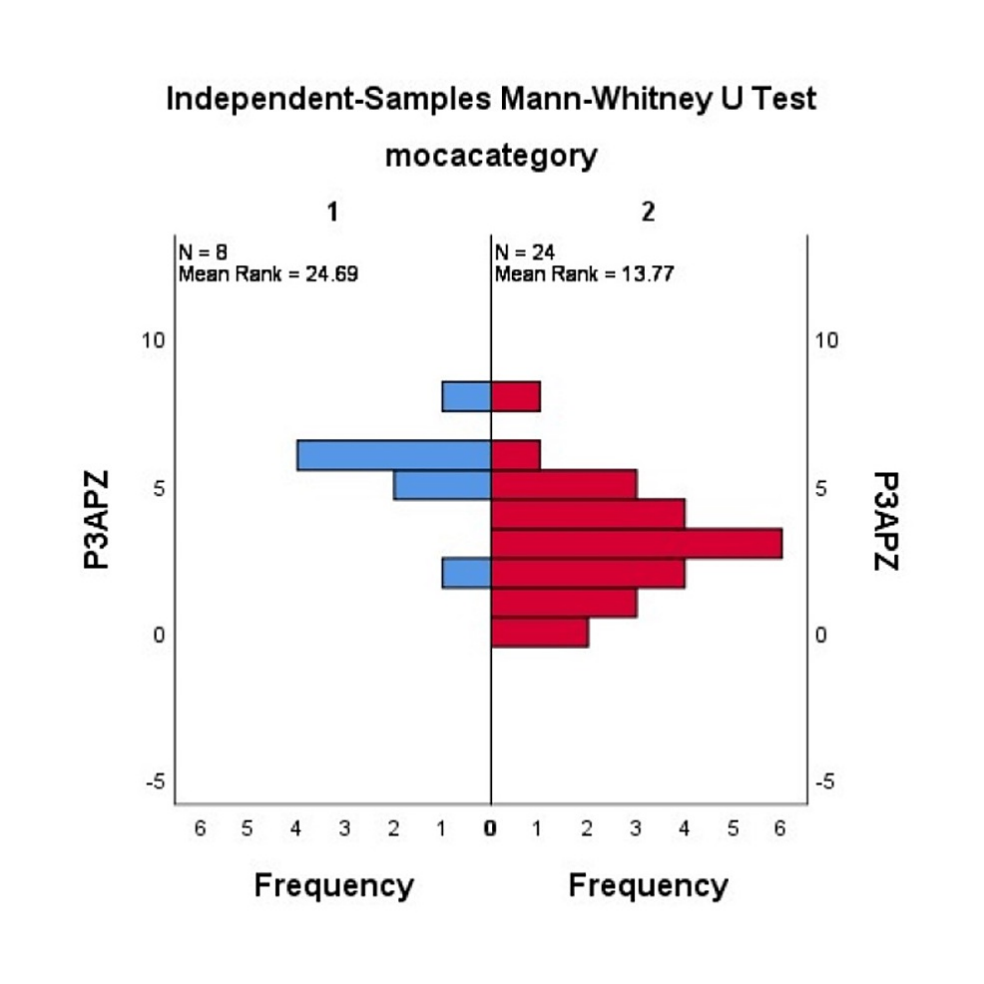
Comparison of MoCA scores with P300 amplitude at Pz using the Mann-Whitney U test MoCA: Montreal Cognitive Assessment; N: number of participants; left blue group: patients with normal cognition; right red group: patients with impaired cognition.

**Figure 2:**
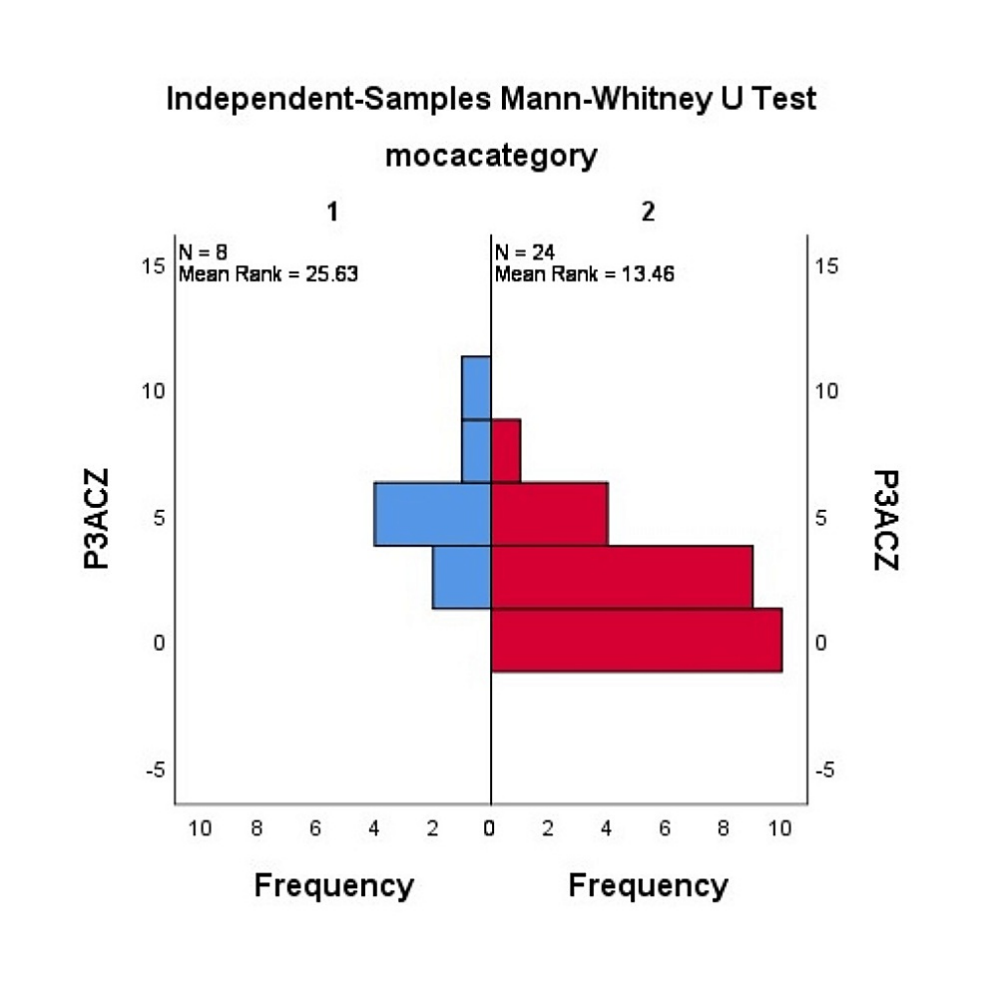
Comparison of MoCA scores with P300 amplitude at Cz using the Mann-Whitney U test MoCA: Montreal Cognitive Assessment; N: number of participants; left blue group: patients with normal cognition; right red group: patients with impaired cognition.

The PD patients were divided into two groups based on their MoCA scores: those with normal cognition and those with impaired cognition. The Mann-Whitney U test revealed a significant difference between PD patients with impaired cognition and patients with normal cognition in the P300 amplitude at Cz (p = 0.001) (Figure 1) and Fz (p= 0.003) (Figure 2). However, the two groups had no significant difference in P300 latency.

## Discussion

The median age of the participants in the current study was 60 years, with an IQR of 13 years (Table 1). Yilmaz et al. and Tang et al. both provided similar data with mean ages of 60.6 and 61.3 years, respectively [15,20]. However, Tokic et al. found that the mean age in their sample was 70.38 years [2]. Dopamine neurons may be susceptible to calcium-mediated neurotoxicity because as they age, they switch from sodium to calcium pacing through calcium channels [4,21].

The male-to-female ratio of the study participants was 1.5:1, with 62% of the male participants and 38% of the female participants (Table 1). Yilmaz et al. also reported the same gender distribution in their study as 62% males and 38% females [20]. Lopes et al. and Tang et al. stated that the number of male participants was 54.5% and the number of females was 45.5% [10,15]. A meta-analysis study by Wooten et al. in 2004 documented that men are about 1.5 times more at risk for PD irrespective of race and location [22].

The present study’s median MoCA score of study participants was 22 and 75% of the study participants had cognitive impairment, as depicted in Table 1. Tang et al. [15] also reported the median score of the late-onset PD group as 22 and in the early-onset PD group as 23.89. Hoops et al. [23] stated that the MoCA score of their participants was 25. The cognitive deficits were noticed among PD patients in the above-mentioned studies using the MoCA questionnaire. To assess the structural, functional, and metabolic correlates of cognitive decline in PD, multimodal imaging methods have been used to investigate the neural basis of cognitive deficits. It has recently been discovered that changes in neurotransmitter systems other than dopamine, such as the noradrenergic, serotonergic, and cholinergic systems, contribute to cognitive loss. The influence of genetic variations on cognitive performance has also been demonstrated. Specifically, polymorphisms affecting the MAPT, COMT, GBA, and APOE genotypes have been linked to cognitive decline [5,24].

Compared with normative data, the amplitude of the P300 wave was markedly shortened (p = 0.000), as shown in Table 3. Similar to our results, Tokic et al. stated that there was a marked reduction in amplitude voltage among patients compared to the reference value [2]. Tang et al. also documented a significant reduction in the amplitude of late-onset PD patients [15]. Similar reports about shortened amplitude in PD patients were documented by Raudino et al. [12].

There was no significant difference between the P300 wave's latency and normative data (Table 2). Both Raudino et al. and Toda et al. provided a similar standard P300 latency report [12,13]. P300 latency was also reported by Goodin et al. to be normal in PD patients who were not demented [25]. In their study, Prabhakar et al. also stated that P300 latency was not prolonged in the early stages of the illness [14]. In contrast to the findings of the aforementioned research, Sarikaya et al., Lopes et al., and Tokic et al. found that the P300 wave's latency was significantly delayed when compared to the control group [2,10,11]. These conflicting results have been highlighted by many authors in their works [12,14]. Given that there is cognitive impairment even in the early stages of the disease, a prolonged P300 latency is anticipated. It is possible that the lack of a perfect control group or normative data is to blame for this contentious conclusion. To standardize normative data for P300 wave components, more research is required.

There was a significant positive correlation between the MoCA score and P300 amplitude (p < 0.05), patients with cognitive impairment had considerably shorter amplitudes than those without impairment (Table 4). Yilmaz et al. [20] reported that P300 latency and amplitude were affected in PD patients with mild cognitive impairment (PD-MCI) than in patients with normal cognition. Also, they stated that P300 provides a diagnostic tool for detecting PD-MCI. Hünerli et al. also reported that P300 amplitude was significantly reduced in the PD-MCI group compared with healthy controls [26].

Limitations

The control group was not taken due to the Institutional Ethical Committee's decision since the research was done during the COVID-19 pandemic situation. Non-parametric tests were used due to a lack of normal distribution of data.

## Conclusions

PD patients showed a significant decrease in P300 amplitude (p = 0.000) but no change in P300 latency. Also, there was a positive correlation between the MoCA score and P300 amplitude (p < 0.05), indicating that if cognition is impaired, P300 amplitude would also be reduced.

Based on P300 potential, practitioners can make an objective assessment of cognition in PD patients and begin supportive treatment for the condition, which can improve the patient's quality of life. Further studies on P300 ERP in patients of PD are needed to implicate ERP usage in the clinical setting.
